# Study on Formation Process and Models of Linear Fe Cluster Structure on a Si(111)-7 × 7-CH_3_OH Surface

**DOI:** 10.3390/ma11091593

**Published:** 2018-09-03

**Authors:** Wenxin Li, Wanyu Ding, Dongying Ju, Ken-ichi Tanaka, Fumio Komori

**Affiliations:** 1 Department of Electronic Engineering, Graduate School of Engineering, Saitama Institute of Technology, 1690 Fusaiji, Fukaya, Saitama 369-0293, Japan; wwenxindiaolong@163.com; 2 School of Material Science and Engineering, Dalian Jiaotong University, No.794, Huanghe Road, Shahekou District, Dalian 116028, China; wanyuD1991@163.com; 3 Advanced Science Institute, Saitama Institute of Technology, 1690 Fusaiji, Fukaya, Saitama 369-0293, Japan; tanaka888@126.com; 4 Ningbo Institute of Materials Industry Innovation, No.12, Lane689, Changxing Road, Jiangbei, Ningbo 315033, China; dyju@sit.ac.jp; 5 The Institute for Solid State Physics, The University of Tokyo, 5-1-5 Kashiwanoha, Kashiwa, Chiba 277-8581, Japan; komori@issp.u-tokyo.ac.jp

**Keywords:** STM, CH_3_OH, evaporation, cluster, linear structure

## Abstract

STM results showed that Fe atoms were deposited on a Si(111)-7 × 7 reconstructed surface, which was saturated with CH_3_OH molecules. Fe atomic linear structure was composed of stable clusters and in-situ observed by the scanning tunneling microscopy (STM). The aim to improve its application of magnetic memory material, both formation process and models, has been explored in this paper. By combining surface images and mass spectrometer data, an intermediate layer model was established. In terms of thermal stability, the most favorable adsorption sites of CH_3_OH were further explored. After that, Fe atoms were deposited on the Si(111)-7 × 7-CH_3_OH surface, forming a linear cluster structure. On the one hand, a new Fe cluster model was put forward in this paper, which was established with height measurement and 3D surface display technology. This model is also affected by the evaporation temperature, which can be consistent with the atomic stacking pattern of face centered cubic structures. On the other hand, the slight height change suggested the stability of linear structures. Even in the condition of thin air introduction, Fe cluster showed a good performance, which suggested the possibility of magnetic memory application in the future. These investigations are believed to have, to a certain extent, increased the probability of forming Fe linear clusters on the surface of silicon substrate, especially according to the models and surface technology we adjusted.

## 1. Introduction

With the rapid development of the smart materials industry, the size of electronic components and devices becomes smaller and smaller [1,2]. Accordingly, thin film materials are given high expectations, such as the regular structure of metal clusters and the high-density of memory units [3,4,5]. In recent years, atomic level technologies have increased the interest of researchers [6]. As the important substrate material, Si(111)-7 × 7 is a surface with well-established atomic and electronic structures [7,8]. Epitaxial growth attaches a great deal of significance to the study of surface properties. This surface is constituted by two types of equilateral triangles, which are referred to as an unfaulted half unit cell and a faulted half unit cell [9]. Along with these triangles, the detailed atomic position theory has been established [10]: Adsorption sites are divided into the center sites, corner sites and rest sites. In this paper, the meticulous preparation of a regular structure is desired not only for the fundamental studies, but also for the application of high-density magnetic memory thin film. Magnetic units are adsorbed on the surface of substrate, forming a stable arrangement. Taking advantage of strong magnetism, the atomic structure of Fe was considered in this study.

In the past few decades, using scanning tunneling microscopy (STM) and first-principles calculation, the adsorption sites of metal atoms on Si(111)-7 × 7 surface have been systematically investigated and identified [7,11]. However, since several specific models were not clear, the formation process could not be controlled accurately. Firstly, in the absence of an intermediate layer (like alcohol), Fe atom would inevitably react with Si, forming the Fe-Si compound [12,13]. In order to preserve the magnetic property of iron, this paper tended to focus on CH_3_OH [14,15]. Through the mass spectrometer equipment and an external heater, some previous assumptions (such as the saturated ratio) were verified [8,10]. As an intermediate layer which iron was deposited on, the specific adsorption model should be explored more deeply. In contrast with ordinary gas adsorption experiments [14], the thermal stability difference between the different adsorption sites was considered in this paper. Secondly, utilizing the evaporation technology of electron beam evaporation [16,17], metal atoms were expected to form a more stable cluster structure on the surface of Si(111)-7 × 7-CH_3_OH. Unlike the adsorption process of gas atoms, different evaporation temperatures correspond to different stacking patterns. By adjusting the temperature parameter, the most suitable stacking pattern could be found for the formation of Fe cluster. Moreover, by analyzing the height difference in 3D images, a growth model of Fe cluster was established. With a clear adsorption process, this model would greatly improve the formation probability of linear Fe cluster structure. Finally, based on the stability of each cluster, the application of linear structure was further investigated [18]. Unlike accidental discoveries in reference [19,20], the regular formation could be realized, which lays a good foundation for the development of high-density storage ultrathin film [20,21,22].

## 2. Materials and Methods

In our experiments, the specific procedure was divided into two stages. In the first stage, CH_3_OH and Fe atoms were adsorbed and observed by JSPM-4500S ultra-high vacuum scanning tunneling microscopy (STM) system (JEOL Ltd., Akishima-shi, Japan). Experiments were performed in the observation chamber with a base pressure of 1 × 10^−8^ Pa. And the vacuum degree is below the 4 × 10^−8^ Pa in the preparation chamber. At room temperature, the STM images were observed in a constant current mode. The tungsten tip made by an electrochemical etching, was installed in the observing chamber. The single-crystal Si(111) substrate (n-type, ~0.1 Ω cm) was ultrasonically pre-cleaned in acetone, ethanol and deionized water. The preparation chamber of the STM system is equipped with three sample holders, which can be used to make three specimens at the same time and conditions. The specific experimental procedures are as follows:

(1) Specimens were degassed in the preparation chamber at about 450 °C. Then, the sample was repeatedly flashed at 1250 °C until a clean well-ordered Si(111)-7 × 7 reconstructed surface was obtained. Afterwards, the sample was cooled down to room temperature in the preparation chamber.
(2) Three Si(111)-7 × 7 samples were successively sent into the observation chamber, which was filled with CH_3_OH gas. At the same time, the mass spectrometer was turned on to monitor the gaseous ion composition in the observation chamber. Si(111)-7 × 7-CH_3_OH samples were heated in 0.2 A current with an external power source. Under the detection of an infrared radiation thermometer, the temperature of samples with heating time in 2, 3 and 4 s, were about 37, 55 and 76 °C, respectively.
(3) After the data of the mass spectrometer was collected, Si(111)-7 × 7-CH_3_OH samples were moved to the preparation chamber. In the STM system, metal atoms can be deposited by controlling the evaporation time and pressure. Through the evaporation device, Fe atoms were evaporated by heating a W filament with iron wire (purity > 99.995%). Different wire dimensions correspond to different rated operating temperatures. Aimed to improve the evaporation temperature in references (about 1000 °C) [20,21,22], a thinner Fe wire was selected to realize a higher temperature (about 1300 °C) and reduce the influence on the linear structure.

The second stage of our experiments focused on the chemical stability of linear Fe cluster structure on the surface of Si(111)-7 × 7-CH_3_OH. Three Si(111)-7 × 7-CH_3_OH-Fe samples were transferred into a composition test chamber, tested by the GammadataScienta SES-100 X-ray photoelectron spectroscopy (XPS) system (Uppsala, Sweden). Additional experimental conditions of them were: (a) none; (b) the introduction of O_2_, keep in 10^−4^ Pa, 10 min; (c) the introduction of thin air, keep in 10^−^^1^ Pa, 2 min. In order to improve the signal-to-noise ratio of data, the area of XPS measurement was kept as 0.1 mm in diameter for all tests. Finally, using the MPMS-7T device, (Quantum Design Ltd., San Diego, CA, USA), magnetization measurements were carried out. A magnetic comparison was made between the normal Si(111)-7 × 7-CH_3_OH-Fe sample (without heating) and our new sample (heating 4 s).

## 3. Results and Discussions

### 3.1. Process and Adsorption Sites of CH_3_OH

**Figure 1:**
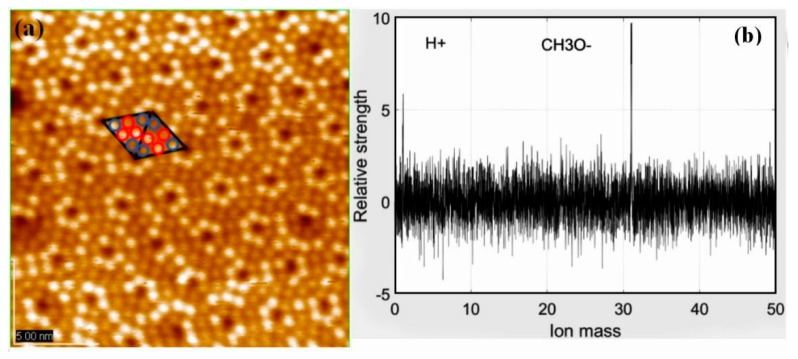
(**a**) STM image of the Si(111)-7 × 7 surface with CH_3_OH concentration is 10^−6^ Pa, and the gas adsorption time is 30 s. The red represents center sites and the blue represents corner sites; (**b**) Mass spectra images of CH_3_OH.

Although much research has been devoted to pure Si surface, little attention paid to its intermediate layer [12,13]. In the case of adsorption of alcohol, the precursor state is equally formed in the faulted and unfaulted halves at room temperature. By analyzing surface images, the ratio of occupied Si adatoms to unoccupied Si adatoms becomes finally 1:1 in the saturated adsorption of methanol [8]. In Figure 1a, according to the analytical method mentioned above, each triangle on Si(111)-7 × 7 surface is composed of six adsorption sites. Further, every six sites can also be divided into 3 center sites (red) and 3 corner sites (blue). Based on the real-time monitoring data of mass spectrometer, the gaseous ion composition was simultaneously observed (Figure 1b). There is a dissociation process in the adsorption stage of methanol gas:

(1) $${\mathrm{CH}}_{3}{\mathrm{OH}=\mathrm{CH}}_{3}O^{-}+H^{+}$$

**Figure 2:**
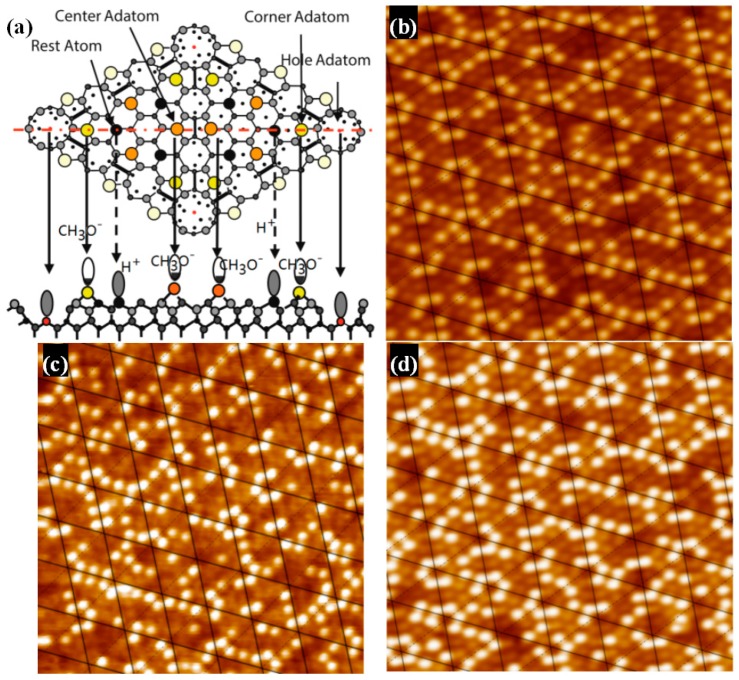
(**a**) The adsorption positions of CH_3_O^−^ and H^+^. The bright spots (CH_3_O^−^) of three Si(111)-7 × 7-CH_3_OH samples was counted; with the heating time is, (**b**) 2 s, center/corner = 86/140 = 0.614; (**c**) 3 s, center/corner = 77/144 = 0.535; (**d**) 4 s, center/corner = 65/133 = 0.489.

Not only STM image of adatoms but also the tunneling spectroscopy at the rest atom position suggests that the CH_3_OH molecule dissociates in a Si adatom to form Si-OCH_3_ and in a rest Si atom to form Si-H, respectively [10]. These facts indicate that the dissociation of alcohol is accomplished via a precursor state in each half unit cell: That is, each half unit cell works independently as if it was a molecule. By observing the relative intensity data of ions, it can be found that the chemical reaction efficiency of H^+^ and Si is much higher than that of CH_3_O^−^. This important feature proves that in the case of the top surface adsorption sites (namely, center sites and corner sites) being occupied, H^+^ can only be adsorbed on the lower rest sites. As a result, a specific adsorption model of methanol is realized (Figure 2a), showing the adsorption location in detail. According to the sample resistance (about 1 KΩ), surface temperature will rise rapidly when the external current passes. Three Si-CH_3_OH samples (like Figure 1a) were heated over different lengths of time. Just as Figure 2b–d shows, two types of adsorption sites were counted: The ratio of center/corner sites decreased with increasing heating times. Under the condition of transient heating above, it can be found that the thermal stability of CH_3_O^−^ in a corner site is stronger than that in a center site. Repeated over another 3 samples, the same ratio change was found. Regularity is the basis of linearity. If the trend of this change is grasped effectively, then the linear structure later should be more obvious. Therefore, it is very significant to verify the change of Si(111)-7 × 7-CH_3_OH before and after transient heating.

### 3.2. Explore the Formation Process and Models of Fe Cluster

**Figure 3:**
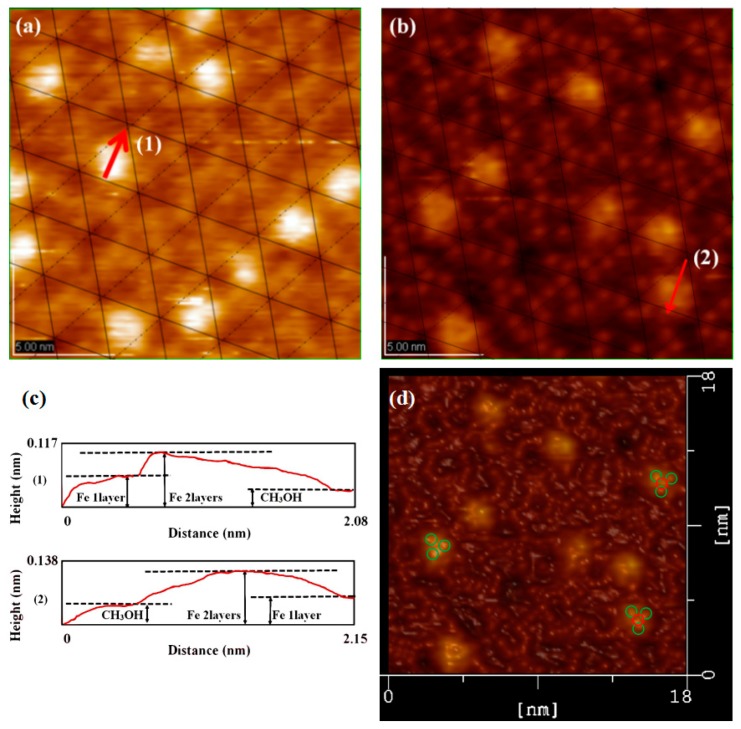
STM images of the Si(111)-7 × 7-CH_3_OH-Fe surface with the iron adsorption site ratio of, (**a**) center/corner = 44/48 = 0.916; (**b**) center/corner = 41/65 = 0.63; (**c**) The height measurement image of the Si(111)-7 × 7-CH_3_OH-Fe; (**d**) is the 3D model of (**b**); The green circle represents an iron atom of the first layer, and the red circle represents an iron atom of the second layer.

**Figure 4:**
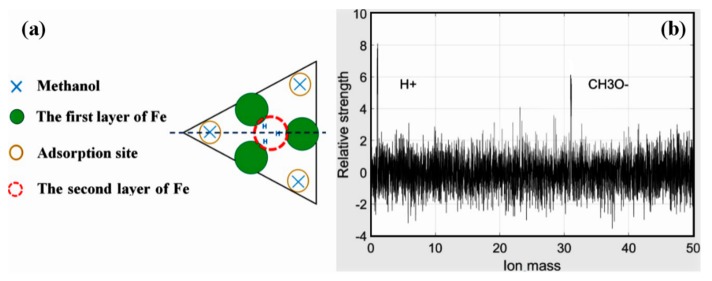
(**a**) Stacking model of Fe cluster; (**b**) Mass spectra images of CH_3_OH.

The structure of Si(111)-7 × 7-alcohol-Fe has attracted wide attention because of its linearity [20,21]. As the degree of Fe atomic adsorption increased, some clusters linked in straight chains. It is noteworthy that the straightly linked chain looks to grow to the lower or upper terrace on the Si(111)-7 × 7-CH_3_OH surface by crossing the step edges, but the low success rate and fuzzy formation process have always puzzled researchers [20]. Attention was again focused on the corner site and center site in order to answer the question: Which one is more favorable for the formation of Fe cluster? The adsorption probability of Fe on center sites should be improved because of the more unoccupied sites. On the basis of Figure 2c,d (heating 3 s and 4 s), Fe atoms were deposited on these two Si(111)-7 × 7-CH_3_OH samples. Just as Figure 3a,b shows, iron was more and more inclined to be adsorbed in the central area of each triangle. Fortunately, several linear structures were also observed on Si(111)-7 × 7-CH_3_OH surface. Additionally, height measurements are presented in Figure 3c. There are three height values (low, high and medium high) which clearly represent: CH_3_OH on the corner sites, the second layer of Fe atoms, the first layer of Fe atoms. In addition to the height data, 3D surface display technology of the STM system was further used to observe the specific structure. In the ordinary scanning image, the adsorption situation was usually analyzed by the spot condition. However, 3D images transformed by the first-principles calculation can show the surface topography in a more visible pattern. Its calculation process is purely based on the positions of atoms, without any other empirical or semi-empirical parameters. If CH_3_O^−^ on corner sites can be considered as an outer triangle, Fe atoms in the first layer are more like an inner triangle, just as Figure 3d and Figure 4a show. Moreover, Fe atoms in the second layer are mostly on the center of both the outer triangle and the inner triangle. On the other hand, when the external power source heated the Si-CH_3_OH samples, data of mass spectra were simultaneously observed. In contrast to Figure 1b, the strength of H^+^ is obviously higher than that of CH_3_O^−^. For the lower position H^+^, it is more susceptible to heating. H^+^ in the free ionic state came from both center sites and corner sites. However, it can be proved that the free ionic state CH_3_O^−^ mainly comes from the center sites. In the case of the disappearance of H atoms near the center sites, these 3 Fe atoms are more likely to form a cluster.

**Figure 5:**
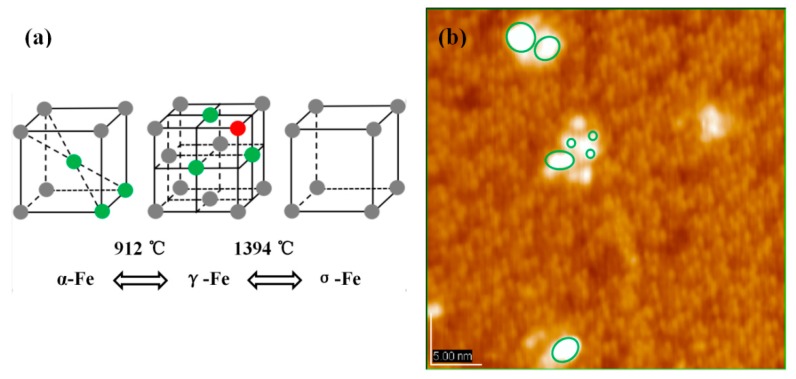
(**a**) Temperature-Structure change diagrams of Fe cluster; (**b**) STM image of the Si(111)-7 × 7-CH_3_OH-Fe surface, which the evaporation temperature is 900 °C.

In addition to the promoting effect of CH_3_OH, high evaporation temperature is another crucial determinant. Just as Figure 5a shows, the structural model of Fe crystal changes with the increase of temperature. Different cluster structures have different stacking patterns, such as face centered cubic unit cell, body centered cubic unit cell, etc. Taking into account the Fe vapor pressure under 900 °C, it is difficult to believe that it is possible to evaporate Fe at such a low temperature. The result given in Figure 5b validates this view: Fe atoms and Fe dots are simultaneously deposited on the surface of Si(111)-7 × 7-CH_3_OH. Unlike the cluster, the dot does not have a regular adsorption structure. If only the atomic parts are observed, they are indeed somewhat similar to α-Fe model in Figure 5a. Compared to the equilateral triangle model of γ-Fe, these Fe atoms present an isosceles triangle arrangement like α-Fe model. A higher temperature was therefore selected to ensure that iron can be evaporated in the form of single atoms. When the temperature is increased to 1200 °C, the arrangement of atoms changes to the γ-Fe pattern. One Fe atom (green one) continues to be adsorbed on the center of the first layer of Fe atoms (red ones), forming the second layer. Results given in different evaporation temperatures support our new model above, where the fuzzy formation process of Fe cluster can now be clear.

### 3.3. Explore the Model and Stability of Linear Structure

**Figure 6:**
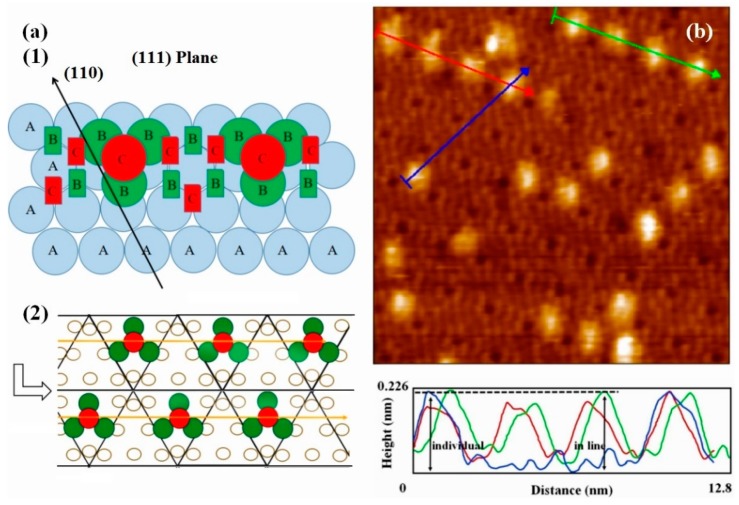
(**a**) The stacking model on the Si(111)-7 × 7 surface, in which, A represent the substrate atoms, B represent stacking atoms in the first stage, C represent stacking atoms in the second stage; (**b**) STM image of the Si(111)-7 × 7-CH_3_OH surface steamed with linear Fe clusters, and its height measurement.

In order to explore the formation process, the surface reaction has been explained by assuming an appropriate activation complex reflecting the potential energy surface. This idea was proposed by the deposition of metal on clean Si(111)-7 × 7 surface and Si(111)-7 × 7-alcohol surface [13], but hasn’t been confirmed yet. The potential barrier theory was used to explain the reaction rate, while the specific reaction characteristics could not be predicted from it. Based on the cluster model above, this paper further explored the linear structure from the point of macro view. In the direction of Reference (111), the atomic stacking model of face centered cubic unit cell (Figure 6(a1)) should be referenced. In atomic stacking theory, as the concentration of iron increases, stable clusters are always presented in the form of intervals. The more regularity, the higher forming probability of linear cluster structure. Just as Figure 6(a2) shows, on the one hand, the interval between units (clusters) can avoid some of the mutual interference problems associated with the small size effect and the surface effect [23,24]. On the other hand, the regularity of each stable cluster further implied the chemical stability of the whole linear structure. During repeated experiments, more and more linear structures (like Figure 6b) were realized. Obviously, there is a continuity between the previous theoretical predictions and experimental data [25]. The stability of Fe linear structure was then preliminarily investigated in height measurements. In a linear Fe cluster structure, it seems that the interaction between each unit could not affect the stability of our cluster model. A single Fe cluster was also compared with the cluster in the linear structure, no obvious height difference was found.

**Figure 7:**
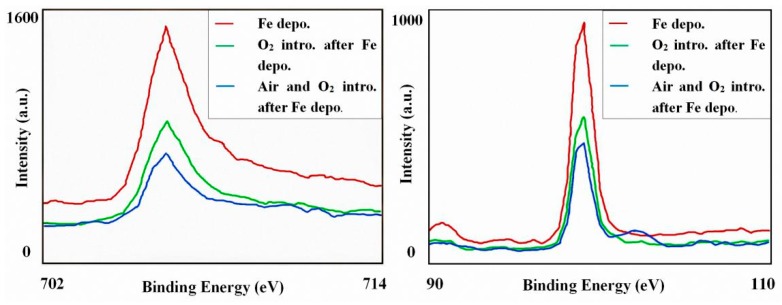
High-resolution XPS spectra of Fe 2p_3/2_ and Si 2p before and after introduction of O_2_ (10^−4^ Pa, 10 min) and thin air (10^−1^ Pa, 2 min).

**Figure 8:**
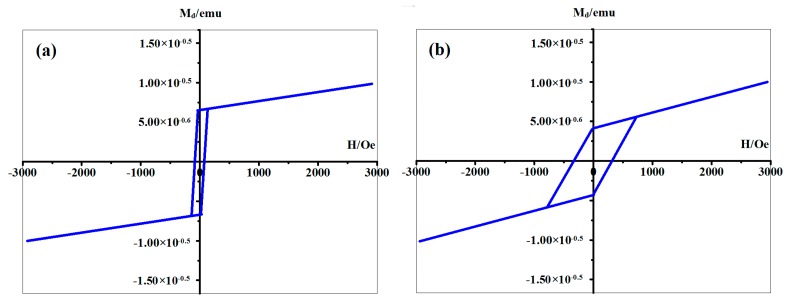
The magnetic test results of the samples, (**a**) the normal Si(111)-7 × 7-CH_3_OH-Fe sample without the heating; (**b**) our new Si(111)-7 × 7-CH_3_OH-Fe sample, with the 4 s heating time on the basis of Figure 6b.

Because of the volatility, the thermal stability of CH_3_OH was studied above. Furthermore, the chemical stability of linear structure should also be detected since the oxidizability of iron. The spectra of XPS were shown in Figure 7. The red line represents the Fe deposited sample, providing the existence of a pure Fe cluster. The peaks of Fe 2p_3/2_ and Si 2p appeared at about 707 eV and 99 eV, which belonged to the Fe-Fe and Si-Si bond respectively. Further, the green line represents the sample after O_2_ introduction and the blue line represents the sample which was exposed in thin air. Based on the XPS results, one conclusion could be deduced that the pure Fe clusters are stable in the above mentioned thin-air condition at room temperature. It can be also proved that the oxygen reacts with Si, instead of Fe. Originally, even if a sample has a perfect regular CH_3_OH layer, the Fe cluster positions would be random, and there might not be cluster chains. However, the driving force making linear structures in Figure 3 and Figure 6 should be the magnetic force of pure Fe clusters. Without improving the formation process, the magnetic strength of the sample is very weak (Figure 8a) and no linear structure can be formed. Accordingly, when linear structures appeared on the surface of our Si(111)-7 × 7-CH_3_OH-Fe, magnetic strength increased obviously (Figure 8b). Therefore, if the iron cluster in the linear structure can be regarded as a relatively independent magnetic unit, it will undoubtedly greatly enhance the existing storage density. From the current situation, new models and methods proposed in this paper have certain advantages in the exploration of a high-density magnetic memory device [26,27].

## 4. Conclusions

The development of high density magnetic memory (film) materials undoubtedly has a significant value in the application market [28,29]. On the surface of Si(111)-7 × 7, a great deal of work is required to promote the structural properties of iron. Originally, CH_3_OH was used as an intermediate layer to prevent the Fe atoms from reacting with silicon, reserving their strong magnetic property. Although the previous linear structures were very attractive, most of them appeared accidentally in the ladder region of surface. The question of how to improve the stability of linear Fe cluster structure has become the focus of our work in recent years. Firstly, the thermal stability of methanol was used to adjust the adsorption ratio of center/corner sites. Afterwards, an intermediate layer model could be established to find the most favorable adsorption sites of Fe clusters. Furthermore, with the increase of evaporation temperature, a double-layer cluster model was successfully found. In the corresponding temperature range, a new structural model of the Fe clusters is basically consistent with the situation of face centered cubic unit cell. Finally, to speed up the pace of application, the regularity of these linear structures was investigated. Results show that the stability of Fe cluster is perfect. Even in the condition of thin air, the performance of our samples is perfect. Overall, the stable Fe cluster improves the unit magnetic strength, which in turn promotes the formation of linear structures. In the future, some nitriding experiments will be explored on the existing Fe clusters, thus greatly enhancing the magnetic properties of the memory units. Metal nitride has robust bonding between metal and nitrogen atoms. Based on our current results, the linear Fe cluster structure will hopefully synthesize the strong magnetic FeN_x_ particles with 5 nm of critical size in the future [30,31].

## Abbreviations

STM	Scanning tunneling microscopy
XPS	X-ray photoelectron spectroscopy
